# Eye Tracking and Visual Arts. Introduction to the Special Thematic Issue

**DOI:** 10.16910/jemr.13.2.1

**Published:** 2022-07-06

**Authors:** Raphael Rosenberg, Rudolf Groner

**Affiliations:** University of Vienna, Austria; University of Bern,, Switzerland

**Keywords:** Eye movement, eye tracking, art, visual arts

## Abstract

There is no visual art without the eye, just like no music without the ear. Visual art does not happen in the eye, but it has to go through the eye. Even for artworks with little visual focus, as in Conceptual Art, we need eyes to create and receive them. In order to see we need to move our eyes. It is therefore not surprising that, for centuries, the eye and its movements have been a major topic of literature on art. It is equally unsurprising that along recent technological improvements of eye tracking, this technology has become prolific for studying visual arts. This special issue of the Journal of Eye Movement Research is the first platform that provides a broad picture of recent developments in this area. In this introduction we present a history of eye movement in art literature, followed by a sketch of some of the oculometric parameters used for studies of visual art. In the third section we showcase each contribution to this special issue.

## 1. The history of eye movements in art literature

Eye movements are a major topic of art literature—from sixth century Byzantium to contemporary academic art history. Over the course of one and a half thousand years there have been four different modes of writing about eye movements which have historically followed one another in a cumulative way: new modes were added, while the older ones remained in use.

### a) Since Antiquity: eye movements indicate the attention of spectators

The relation between visual attention and the direction of the eyes must have been observed since time immemorial. Written evidence can be traced back to classical antiquity (48, pp. 35-36). As early as in the second century CE, Achilles Tatius discusses eye movements of beholders when describing a city and its buildings (46, E, 1-5). At around 553 CE, Procopius of Caesarea gives an account of the Hagia Sophia in Constantinople. It is the oldest known detailed description of an existing building. In regard to the dome of the church, Procopius first enumerates architectural parts and then describes the eye movements of the beholder:

[…] *each detail attracts the eye and draws it on irresistibly to itself. So the vision constantly shifts suddenly, for the beholder is utterly unable to select which particular detail he should admire more than all the others. But even so, though they turn their attention to every side and look with contracted brows upon every detail, observers are still unable to understand the skilful craftsmanship but they always depart from there overwhelmed by the bewildering sight.* (29, I, i, 48-49).

Procopius uses the confusion of the eye to stress an essential element of the Hagia Sophia: seen from the inside, the huge dome appears to be supported by thin walls which are pierced by arcades and windows. We can assume that the architects wanted the visitors to wonder how this is physically possible (the buttresses of the dome are only visible from the outside).

### b) Since the Renaissance: eye movements as source of aesthetic pleasure

In the city of Florence fundamental changes occur in the visual arts around the year 1420. We subsume them as the beginning of a new epoch—the Renaissance. In architecture, round arches and classical order replace Gothic pointed arches; the invention of geometric perspective revolutionises painting; the anatomy of the body becomes the scale for sculpture. These changes are accompanied by a significant increase in the written discourse on art and by a higher status for artists, who can now claim to be guided by theory.

In this growing discourse on visual arts, eye movements take an innovative role: they express and legitimize aesthetic qualities. The oldest example known to us dates from the 1460s. In his *Treatise of Architecture,* the Florentine artist Filarete compares round and pointed arches. As can be expected, he argues for the superiority of the round arch used by ancient Romans, by Filarete himself, and by all fellow 'Renaissance' architects. Remarkably, Filarete does not appeal to historical arguments, but justifies the superiority of the round arch with the movement it induces in the eye:

*It cannot be doubted that nothing which impedes the sight in any way is as beautiful as the one that leads the eye rather than restraining it. Such is the round arch. As you have noticed, your eye is not arrested in the least when you look at a half-circle arch. […] The pointed is not so, for the eye, or sight, pauses a little at the pointed part and does not run along as it does on the half circle* (10, pp. 230-231, translation by the authors).

Filarete's reflection combines two thoughts: First, he assumes that the eye follows the form of an artwork. Secondly, that beauty correlates with the pleasure caused by the movement of the eye. Large curves over which the eye can glide unhindered are more beautiful because they lay out a more pleasant course to the eye than angles and turns. In one of the very first eye tracking experiments, George Stratton has shown that there is no physiological evidence for these assumptions (45). Nonetheless, in Early Modern Times Filarete's reasoning was extremely successful. Three centuries later, it even forms the basis for William Hogarth's glorification of the serpentine line in a bestseller of 18^th^ century aesthetic theory:

*The eye hath this sort of enjoyment in winding walks, and serpentine rivers, and all sorts of objects, whose forms, as we shall see hereafter, are composed principally of what, I call, the* waving *and* serpentine *lines.* (19, p. 25)

Filarete’s text was not printed until modern times and never gained a large readership. It is difficult to say whether later authors expressing similar assumptions (i.e.^40^, p. 141) read his treatise or came up with the same idea independently. The metaphor seems to have spread during the later 17^th^ century, most likely due to the work of André Félibien. In the service of Louis XIV, we can consider him the first professional art historian and he also had a major role in the development of art description (35, 36). Félibien often uses eye movements to justify that paintings are well designed:

*But to judge this beautiful conduct and effect of colours, we must only consider how they are so judiciously placed in this painting, that the eye passes imperceptibly from one to the other, without finding anything that offends it by too much disproportion or hardness*. (8, p. 27, translation by the authors)

### c) Since the 17^th^ century: the eye follows the composition of the artwork

Just like Filarete, Félibien uses eye movements to justify aesthetic qualities. However, instead of discussing a general form of architecture, he describes specific paintings and explicitly relates eye movements to their composition—to the way the painter distributes figures, objects and colours on the canvas and in the represented space (9, pp. 67, 79, 81-83, 114, 125). In doing so, Félibien established a new idiom that became an integral part of art literature in France and beyond (5, p. 118; 6, pp. 108, 114-115; 19, pp. 25-28, 38-39, 112 and passim; 22, II, p. 66; Piles, 1668, pp. 28, 97, 105, 112; 1677, pp. 112-113, 231-232; 1708, pp. 106-107; 32, p. 229; 33, pp. 58, 61, 67; 47, pp. 109-111).

Artists have of course always paid attention to the distribution of single elements in their paintings. However, the idea of pictorial composition was only defined in the 17^th^ century. André Félibien and Roger de Piles play an essential role in establishing the concept of pictorial composition, which was to become a central category of art theory (31) and both relate the composition of paintings to the movement of the eye.

Following up on this idea, and thus ultimately on Filarete, Denis Diderot postulates in 1767 that in every painting there should be one and only one line of composition and that this line will guide the eye of the viewer (38). The idea of a compositional path to be followed by the beholder’s eye remains popular among art historians both in relation to paintings and sculptures until today.

### d) Since the 19^th^ century: eye Movements and the history of perception

A new dimension was added to this discourse in the middle of the 19^th^ century: the idea that the history of visual arts is driven by a “history of the eye”, i.e. by changes of vision (cf. 37). As early as 1859 Berthold Riehl, professor for cultural history at the University of Munich, writes:

*One might think that sun, air and clouds, water and mountains and trees and rocks have changed their nature over the centuries, that nature itself has changed its style, if we did not know too well that only the human eye has changed its nature in the meantime, that every generation sees in a different style.* (34, pp. 74, translation by the authors)

The assumption of a history of the eye became popular in the early 20^th^ century and had a revival in the 1970s. Among the best-known representatives we can list Alois Riegl, Heinrich Wölfflin, Hermann Bahr, Walther Benjamin, Michael Baxandall, John Berger, and Michel Foucault. However, it is often unclear whether or how much they consider a history of the eye as more than a metaphor. Many of them do not specify the involvement of physiological processes, but some do explicitly postulate correlations with eye movements, assuming for instance that cultural differences in art are correlated to eye movements patterns:

*Italians have an architectural-plastic talent accustoming the eye to trace the form of things, to see each individual figure in space and to ascertain the physicality of a thing by scanning it with the eye [...]. The Italian vision isolates, the vision of Dutch people and Germans connects; the former is used to the mobility of gaze, the latter to the quiet-looking eye* (50, pp. 211f., translation by the authors).

## 2. Oculometric parameter of art perception

Eye movements have been an important topic in the literature on art for centuries. As different as ideas about eye movements could be, authors always assumed continuous movements. Only at the end of the 19^th^ century ophthalmologists realized that eye movements are not continuous but jerky (48, 49): they essentially consist of stop and go. Eye trackers deliver measurements of the position of the pupil (called fixations) and the jumps in-between (called saccades). By analysing those measurements, we can determine the timing and whereabouts of fixations as well as the timing and course of saccades (14). This data delivers insightful information about the processes of making and perceiving of artworks. So far, the vast majority of studies in this field focuses on the reception of two-dimensional pictures, whereas experiments related to the making of art and or three-dimensional stimuli are more difficult to realize. Those limitations also apply to the papers of this special issue.

The essential parameters of eye tracking research are fixations and saccades*. Fixations* are the moments the eye stops and humans are physically able to visually perceive an object. Eye trackers make it possible to locate every single fixation. This delivers information about the whereabouts of attention: what did the person look at, and what did they definitely oversee? Dots and circles can be used to visualize the location of single fixations, heat maps to visualize the density of fixations in the different areas of the stimuli. We can define “areas of interest” (AOI, also called regions of interest ROI) and use them to calculate the number of fixations within and outside of specific areas of artworks. We can set AOIs top down (i.e. comparing some figures of a painting with other figures as 13; 18), in a neutral manner such as a grid (39) or bottom up by using recorded data as the area most often looked at (11)). The analysis of fixations reveals the variable degrees of saliency within and across artworks. We can, for instance, study whether and to what extent this depends on specific properties of the artwork, the viewer, and or the condition of beholding.

The *time of fixations* is insightful on different levels: 1) the cumulated amount of time that people spend looking at certain artwork and or area of artwork is a measure for the interest in the artwork and that area 2) the moment in time when a fixation rests for the first time and or repeatedly in a specific area of the stimulus is a measure for the moment when the viewer discovers and or is particularly interested in this part of the artwork; 3) the duration of single fixations is highly variable – commonly between less than 100 and more than 500 milliseconds (14). The length of a fixation depends on the viewer and significantly increases with age. On the other hand, it is related to cognitive processes: longer fixations indicate a higher cognitive load (12). Therefore, the duration of fixations can depend on the task and the expertise (43; 44) as well as vary in different parts of an artwork (24); 4). It is also revealing to measure changes of the duration of fixations over the entire beholding time. Usually, the duration of fixations changes during the course of beholding pictures. A change related to changes of the amplitude of saccades (see below). Duration might also change in relation to events such as when beholders listen to an audio guide or speak while looking at an artwork.

*Saccades* are quick shifts of the eye from one fixation to the next one. While performing a saccade the eye is basically blind (7). By locating fixations, we know the beginning and the end of a saccade and its length. When using a high-speed eye tracker, we can also determine the course and the speed of saccades. Eye tracking research has largely been focussed on fixations. Analytical tools for visualizing saccades are not yet as developed as those for fixations (21). However, since—as sketched above—art literature so often discusses the *movement* of the eyes, saccades are a prominent topic for art related eye tracking studies. An interesting class of saccades are very small saccades, called *microsaccades* which are related to various processes of perception and cognition (23) and distinguish looking from seeing (20; 42).

Having determined the position of saccades and fixations, we can follow the course of the eye through the stimulus, called a scan path (Noton & Stark, 1971; 17; 41; 51). It shows that actual eye movements are more chaotic than it was assumed in art literature. Eye movements are not smooth, but jumpy, and the eyes of any normal viewer never follow regular curves through the composition of any artwork. At first glance sequences of saccades seem to be erratic. Nonetheless, saccades indicate how viewers relate different parts of an artwork with each other. It is therefore revealing to analyse which fixations are frequently repeated. (2; 39) have shown that often repeated saccades do correlate with the structure, the “composition” of paintings.

The *amplitude of saccades* is also variable. We can measure it either referring to the eye, in visual angles measured in degrees of a circle, or referring to the stimuli, in millimetres or in the number of pixels (when the stimuli are shown on a digital screen). Long saccades imply that the beholder is looking at parts of the stimulus far apart, indicating the beholder is attempting to get an overview of the stimulus. Small saccades indicate that the viewer is more focused on details (25). (4) already noted that there are two successive phases in the viewing of images: First, for a few seconds, global viewing with large saccades and short fixations, followed by local viewing with small saccades and long fixations. The ratio of global versus local gazes thus depends on the course of beholding, but also on the stimulus and its parts, on the expertise of the viewer and any tasks they were asked to perform (43).

Art literature produced numerous assumptions about the direction of eye movements, one of which states that Western painting is fundamentally designed to be viewed from left to right (1). It is therefore interesting to quantify the direction of the saccades. It is yet unclear how much the *direction of saccades* correlates with the subjectively perceived orientation of movements in pictures, whether it be the depicted movement in space, the orientation of depicted gestures or depicted gazes.

In most visualizations, saccades are represented as straight lines between two points symbolizing the fixations, although this is an approximation. Whether and to what extent the exact *course of saccades* correlates with the person, the stimulus, or the circumstances of art viewing, is a yet scarcely explored topic. This is also the case for the *velocity of saccades* that may correspond with the perceived dynamism of artworks (3).

## 3. What do we learn from the articles in this issue?

In his monograph “How People Look at Pictures: A Study of the Psychology of Perception in Art”, the educational psychologist Guy Thomas Buswell (4) presented the recordings of approximately 200 observers looking at different reproductions of artworks. It was the first large empirical study on art and eye movements. Nicholas J. Wade (2020) gives an overview of Buswell’s work and outlines its connections to earlier work of Stratton (45, 1906) and Judd et al. (1905). Buswell’s analysis was graphical rather than statistical, but—similar to the work of Yarbus (1967)—it had great influence on later theoretical and experimental work.

With regard to the composition of paintings, the linear perspective, meaning that all parallel lines converge in a single vanishing point, has been used since the Renaissance to create the illusion of three-dimensional space on the picture plane. The article by Arthur Crucq (2021) “Viewing-patterns and perspectival painting: An eye-tracking study on the effect of the vanishing point” analyses the question whether the gaze of the beholder is affected by the underlying structure of linear perspective. In some compositions the vanishing point appears to attract the eye of the study participant, especially if the vanishing point coincides with the central axis of the painting. The effect is even stronger when the vanishing point converges with a major visual feature of the composition, such as an object or figure. The question remains open what comes first, the location of the vanishing point or the visual feature, especially since the study also shows that if the vanishing point is less salient, it does not attract much attention.

In the article “The role that composition plays in determining how a viewer looks at landscape art” Tanya Beelders and Luna Bergh (2) analyse the scan paths of 65 participants looking at three compositions. Based on a similarity index constructed by using the known intention of the artists, the authors conclude that composition is successful in leading the eye, although the order of fixations varies. It is argued that composition is influential with respect to the location of salient elements, but less so for guiding the sequence of fixations as scan paths.

The article “Does pictorial composition guide the eye? Investigating four centuries of last supper pictures” by Rosa Sancarlo, Zoya Dare, Jozsef Arato and Raphael Rosenberg (39) investigates the saccadic eye movements of viewers looking at 14 paintings representing the biblical scene of the Last Supper. The same persons were later asked to draw the composition lines of those paintings. The authors propose a novel coefficient of similarity quantifying the similarity between the saccades of different observers, the similarity between the compositional drawings of different observers, and the similarity between saccades and compositional drawings. The authors found a statistically significant similarity between the saccades and between the compositional drawings, as well as between the compositional lines of the paintings and the saccades. They therefore conclude that composition does influence visual perception by guiding eye movements.

Regarding styles of visual art, the article “A quantitative analysis of the taxonomy of artistic styles” by Viviane Clay, Johannes Schrumpf, Yannick Tessenow, Helmut Leder, Ulrich Ansorge and Peter König (2020) explores the aesthetic qualities of seven art styles. They use artificial neural networks to extract features and subsequently transform photographs into artificially generated images. In this way they generate new images resembling the style of specific artists. In a visual singleton search study, subjects had to locate a style-outlier image embedded among three images of a alternative styles. Reaction time and accuracy were measured and analysed, revealing significant differences in behavior when viewing images of varying art styles.

In the article “Absorbing the gaze, scattering looks: Klimt’s distinctive style and its two-fold effect on the eye of the beholder” Anna Miscena, Jozsef Arato and Raphael Rosenberg (24) study whether Klimt’s distinctive style juxtaposing realistic features and flattened ornamental patterns causes a specific looking behaviour. While their eye movements were recorded, thirty viewers were shown three groups of portraits comprising of images created by Klimt as well as more abstracted and rather realistic portraits of the same historical period. The recorded data shows that Klimt’s distinctive style induces a specific eye-movement pattern with alternating longer (“absorbed”) and shorter (“scattered”) fixations. This demonstrates a behavioural correspondence to art historical interpretations.

In the context of reading, typography plays an important role, combining subjective aesthetic appreciation with practical aspects like legibility. Familiar letter forms build up legibility after centuries-long exposure. In the article “You read best what you read most: An eye tracking study“ Uroš Nedeljković, Kata Jovančić and Nace Pušnik (2020) examined the legibility in the context of familiarity, whether it is affected by the time of exposure to a particular typeface or by the typeface’s universal structure. Experiments were conducted using new, for this purpose designed typefaces as stimuli. The results confirm that the reader’s familiarity with a typeface indeed influences reading speed. The universal letter structure is the constant that establishes legibility, but the period of exposure to uncommon letter forms has a positive impact on legibility.

In their study “Reading English-language haiku: An eye-movement study of the ‘cut effect’” Thomas Geyer, Franziska Günther, Hermann J Müller, Jim Kacian, Heinrich René Liesefeld and Stella Pierides (2020) present data where study participants were reading three-line haiku that consisted of two (seemingly) disparate parts, a (two-line) ‘phrase’ image and a one-line ‘fragment’ image. How do readers process the conceptual gap between these images when constructing the poem’s meaning? This process is reflected in their patterns of reading eye movements. The predicted ‘cut effect’, defined as an extended fixation time on the fragment line relative to the other lines, was observed. It was demonstrated that the formal-structural and conceptual-semantic properties of haiku were associated with systematic changes in how individual poem lines were scanned during the first reading and then (selectively) resampled in second- and third-pass reading. The cognitive processes during the comprehension of haiku are invoked by both form-and meaning-related features of the poems.

In “Interaction between image and text during the process of biblical art reception” Gregor Hardiess and Caecilie Weissert (18) ask how naive observers without a distinct religious background approach illustration of the Bible that combine image and text. The authors used four images from the book “New biblical figures of the Old and New Testament” published in 1569. Eye movements of participants were measured in order to characterize and quantify the scanning behaviour. The authors show that texts captured attention early in the process of inspection, but text and image also interact. The semantics of texts guide eye movements through the image, supporting understanding of the narrative.

Nino Sharvashidze and Alexander Schütz (43) compare in the article “Task-dependent eye-movement patterns in viewing art” a group of art history students with a group of students having no art education background. Both groups viewed 3 blocks of 12 paintings in which they were each to determine the art movement (Baroque, Cubism, Expressionism, Impressionism, Post-Impressionism, Romanticism), the date, and the medium of the paintings (Oil, Pastel, Watercolour, Acrylic, Chalk and Ink) while eye movements were recorded for five seconds. The statistical data analysis showed that the participants adjusted their viewing strategies according to the task, resulting in longer fixation durations and shorter saccade amplitudes for the medium detection task. In the expert group a higher task accuracy and subjective confidence, less congruence and higher dispersion in fixation locations was found. Expertise also influenced saccade metrics towards larger saccade amplitudes, suggesting a more holistic scanning strategy of the experts in all three tasks.

In “The closer, the better? Processing relations between picture elements in historical paintings” Manuela Glaser, Manuel Knoos and Stephan Schwan (2020) investigate how audio explanations such as those given by museum audio guides influence perception and the cognitive processing of historical paintings. Pairs of spatially close or distant picture elements and their semantic relations were named in an audio text either immediately after each other or with descriptions of other elements in between. It was predicted that the number of backward fixations to the first picture element should be higher if they are spatially close rather than spatially distant. There should also be more backward fixations if the elements are named temporally close rather than temporally distant. Analogous predictions were made for the retention of these picture elements and their relations. A 2x2x2 within-subject design (*n*=36) with spatial distance (close vs. distant), temporal distance (close vs. distant) and painting (by Leutze vs. West) revealed more background fixation counts for spatially close compared to spatially distant elements but only for one of the paintings. The relations between the spatially close pairs were remembered better than between the spatially distant pairs. The results are discussed in the context of multimedia learning and text coherence.

Eye tracking research in art viewing has usually been conducted in a laboratory setting where reproductions must be used in place of original artworks and the viewing environment is of course very different from a museum. Due to recent technological developments it has become possible to run studies on-site in exhibition venues. Three studies were conducted in different museums with different technologies. In their article “Testing a calibration-free eye tracker prototype at the Kunsthistorisches Museum in Vienna” Zoya Dare, Hanna Brinkmann and Raphael Rosenberg (2020) employed a prototype of a calibration-free remote eye tracker hidden below selected paintings. This allowed to analyse the time spent by viewers looking at the paintings, showing that certain paintings consistently drew significantly more prolonged attention. While the data quality from the eye tracker prototype was not sufficient to conduct an in-depth analysis of within-painting gaze movements, this study serves as an interesting step towards an unobtrusive examination of the art viewing process of museum visitors.

The study by Salma Mesmoudi, Stanislas Hommet and Denis Peschanski (2020) titled “Eye-tracking and learning experience: Gaze trajectories to better understand the behavior of memorial visitors” evaluated the potential of mobile eye-tracking to quantify visitors’ experience and behaviours during the visit of the "Genocide and Mass Violence" area of the Caen memorial. Eye-tracking data of 17 visitors were collected. The viewing time each visitor spent in front of 19 selected regions of interests was analysed and compared. This allowed the comparison of the gaze trajectory together with the information about regions of interests and to identify certain behaviours, such as avoidance. Clustering analysis revealed some typical trajectories performed by specific subgroups.

In 2018, the Austrian Gallery Belvedere rearranged its permanent collection. Luise Reitstätter, Hanna Brinkmann, Thiago Santini, Eva Specker, Zoya Dare, Flora Bakondi, Anna Miscená, Enkelejda Kasneci, Helmut Leder, Raphael Rosenberg (2020) seized this opportunity to investigate possible changes in the viewing behaviour before and after the museum’s restructuring. The authors of the paper “The display makes a difference: A mobile eye tracking study on the perception of art before and after a museum’s rearrangement” employ a mixed-method approach combining mobile eye tracking, subjective mapping (a drawing task in conjunction with an open interview), and a questionnaire in order to relate gaze patterns to processes of constituting meaning. The results show that the new display did make a difference: it generally increased the viewing times of the artworks, extended the reading times of labels, and deepened the visitors’ engagement with the artworks in their reflections about the exhibition. In contrast, however, interest in specific works, in forms of art, and preferences remained robust and independent of presentation arrangement.
